# Draft Genome Sequence of *Deinococcus xibeiensis* R13, a New Carotenoid-Producing Strain

**DOI:** 10.1128/genomeA.00987-13

**Published:** 2013-12-05

**Authors:** Yaochi Hu, Xian Xu, Ping Song, Ling Jiang, Zhidong Zhang, He Huang

**Affiliations:** State Key Laboratory of Materials-Oriented Chemical Engineering, College of Biotechnology and Pharmaceutical Engineering, Nanjing University of Technology, Nanjing, People’s Republic of Chinaa; Institute of Microbiology, Xinjiang Academy of Agricultural Sciences, Urumqi, Xinjiang Uigur Autonomous Region, People’s Republic of Chinab

## Abstract

*Deinococcus xibeiensis* strain R13, isolated from radiation-contaminated soils, synthesizes a unique ketocarotenoid, deinoxanthin. Here, we present a 3.49-Mb assembly of its genome sequence, which can help us find the key genes of the deinoxanthin biosynthesis pathways and modify genes obtaining a high yield of the new carotenoid.

## GENOME ANNOUNCEMENT

Carotenoids are a family of yellow-to-orange-red terpenoid pigments synthesized by many microorganisms. They are commercially used as colorants, feed supplements, and nutraceuticals in the food, medical, and cosmetic industries (1, 2). *Deinococcus radiodurans* is a Gram-positive red-pigmented bacterium that was originally identified as a contaminant of irradiated canned meat (3). It is extremely resistant to a number of agents and conditions that damage DNA, including ultraviolet (UV) radiation and exposure to hydrogen peroxide. *D. radiodurans* synthesizes a unique ketocarotenoid, deinoxanthin, as its major carotenoid (4, 5). Deinoxanthin exhibits a significantly stronger reactive oxygen species (ROS)-scavenging ability than other known carotenoids, such as lycopene, β-carotene, and zeaxanthin (6), and the strong antioxidant effect of deinoxanthin plays an important role in the radioresistance of *D. radiodurans*.

We recently characterized a new bacterial strain, *Deinococcus xibeiensis* R13. Strain R13 is an aerobic, Gram-positive, red-pigmented coccus with gamma-radiation resistance to >10 kGy and UV resistance to >700 J · m^-2^ (7). R13 produces deinoxanthin, and its color is deeper than that of *D. radiodurans*. It is possible that the genes and enzymes of its carotenoid biosynthesis pathways are different from those of *D. radiodurans*. Besides, the molecular mechanisms of deinoxanthin for the extraordinary resilience of this species remain poorly understood. Therefore, an investigation of the genetic information and characteristics of *D. xibeiensis* R13 is urgently needed. Gene analysis can help find the key genes of the deinoxanthin biosynthesis pathways and modify the genes obtaining a high yield of the new carotenoid.

Here, we present the draft genome sequence of strain R13 using the Illumina MiSeq platform, which was performed by Shanghai Personal Biotechnology Co., Ltd., with a paired-end library. A total of 349,223,286 reads and 1,691,024 Illumina reads, totaling 612 Mb, were assembled using Newbler software, resulting in 298 contigs with an N_50_ of 14,748 bp (8). The largest contig assembled is 81,857 bp. After combining all of the contigs, 294 scaffolds with a genome of 3,288,779 bp were generated using SSPACE software (9). Gaps between scaffolds and within scaffolds were closed by GapCloser. The G+C content is 66.53%. Gene prediction and annotations were performed by the programs Glimmer 3.0 and Blast2GO. A total of 3,279 protein-coding sequences (CDSs) were predicted from the draft genome. The draft genome has 2 rRNA operons and 45 tRNAs predicted by RNAmmer (10) and tRNAscan (11), respectively.

A total of 1,771 proteins were mapped onto Kyoto Encyclopedia of Genes and Genomes (KEGG) pathways, and 47 of these genes were related to the metabolism of terpenoids and polyketides. In addition, the R13 genome contains genes involved in carotenoid biosynthesis, such as genes encoding geranylgeranyl diphosphate synthase (*crtE*) at contig 214, phytoene synthase (*crtB*) and phytoene dehydrogenase (*crtI*) at contig 87, and lycopene cyclase (*crtLM*) at contig 10. The genome sequence of strain R13 provides a genomic basis for in-depth comparative genome analyses to understand the specific mechanisms of radioresistance and the deinoxanthin biosynthesis pathways.

### Nucleotide sequence accession numbers.

This whole-genome shotgun project has been deposited at DDBJ/EMBL/GenBank under the accession no. AXLL00000000. The version described in this paper is version AXLL01000000.
